# Death, taxes, and rhomboids: Understanding the ubiquitous roles of the rhomboid protein superfamily

**DOI:** 10.1016/j.jbc.2025.110699

**Published:** 2025-09-12

**Authors:** Henry Sawczyc, Spyridon Kosteletos, Adam Lange

**Affiliations:** 1 Research Unit Molecular Biophysics, Leibniz Forschungsinstitut für Molekulare Pharmakologie (FMP), Berlin, Germany; 2 Institut für Biologie, Humboldt-Universität zu Berlin, Berlin, Germany

**Keywords:** rhomboid, intramembrane proteolysis, ERAD, membrane protein, structural biology

## Abstract

The rhomboid superfamily is the largest family of membrane proteins, containing over 122,000 members (both active and inactive proteases) across nearly all domains of life. The high number of members, as well as the conserved roles undertaken by members, indicates an ancient origin and nature of function. However, the high structural similarity and multiple active homologs per species or cell have made specific functional characterization difficult. Where function is known, members appear to be not imminently necessary for life but organizational or housekeeping in nature. Historically, active protease members have been the focus of research because of the ease of biochemical characterization through monitoring proteolytic cleavage. The active members appear to possess conserved and specific recognition motifs for substrates, although no consensus sequence for substrates exists. Instead, substrate access and recognition appear to occur through recognition by dynamics. In recent years, bioinformatic work has shifted focus toward catalytically inactive members and the functional characterization of these numerous but often forgotten “dead” proteases. These inactive proteases are now known to play key roles in the recognition and retrotranslocation of poor-quality membrane proteins. Recent work on the rhomboid-fold’s unique ability to thin the lipid bilayer has enhanced mechanistic knowledge of both inactive and active protease function. Due to the ubiquitous presence of rhomboid members and their implications in a wide range of disease states, they are high-priority pharmaceutical targets; however, development of specific inhibitors has been hampered by the tight conservation of both the active site and the common rhomboid fold.

The rhomboid protein superfamily, named for the first characterization in fly mutation screens (1), has since expanded to cover a large number of proteins across all domains of life (Fig. 1). The functional roles of these proteins are varied (Table 1) and span beyond the active protease role initially assigned to the namesake Rhomboid-1 in *Drosophila melanogaster* (2, 3). Members have been discovered in numerous essential roles, such as growth factor signaling (4, 5), protein quality control and turnover (6, 7, 8), as well as key players in parasitic invasion and life cycle (9). Although sequence conservation between members is low (10), nearly all members found so far share a common central fold (the “rhomboid fold”): a compact six-transmembrane (TM) α-helical bundle (11). This low sequential but high structural homology is often found in membrane proteins because of the nature of the hydrophobic environment and the altered evolutionary pressure for helix packing within the membrane compared with the evolutionary pressures for soluble proteins (10). Whilst this core structural motif and several short sequences within this fold are highly conserved, some members, primarily eukaryotic, have obtained an additional TM helix throughout evolution (denoted either as 1+6 or 6+1 depending on whether the addition is N or C terminal) (10). Others have gained soluble domains, such as the model *Escherichia coli* rhomboid protease GlpG with an N-terminal (cytosolic) soluble domain (12), or a combination of both additions. Overall, the common fold has led to relative ease in identification of family members through bioinformatic searches, and the biochemical work in the past 25 years has shed light on the functional roles of these members, primarily for the active proteases (for a brief overview, please see Table 1 as well as other reviews focusing more on rhomboid function (12, 13, 14)). However, much work is left to characterize these members aside from their genetic sequence and common fold.

**Figure 1:**
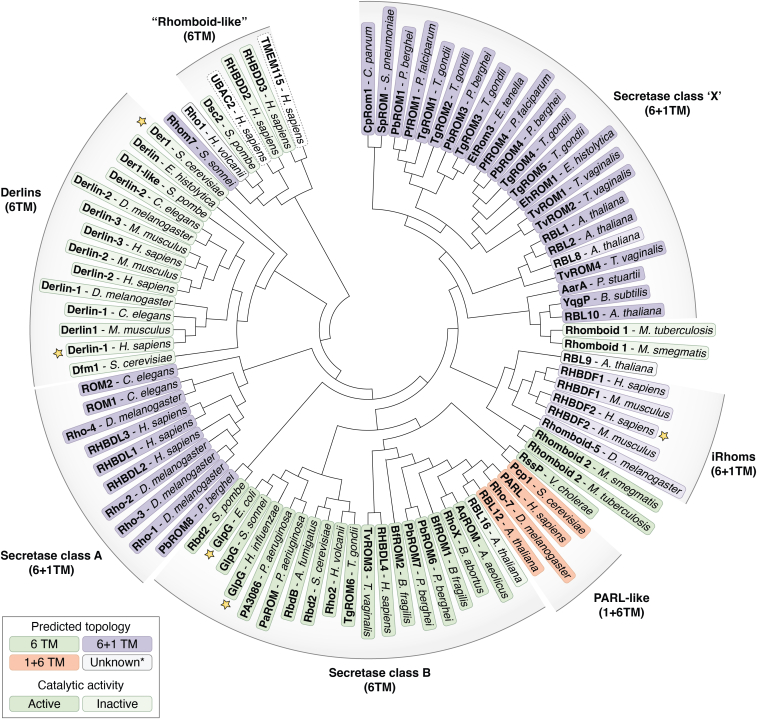
**Phylogenetic tree of biochemically or functionally characterized rhomboid proteins found in the literatureplotted using neighbour-joining tree function after whole-sequence alignment.***Green*, *purple*, and *orange* colorations denote 6TM, 6+1TM, and 1+6TM topology, respectively (either from the literature or as predicted by TOPCONS (158), where not available). Exceptions to this are UBAC2, TMEM115 (both from *Homo sapiens*), RBL9 (from *Arabidopsis thaliana*), and Rho1 (from *Haloferax volcanii*), where TOPCON shows a large deviation from the expected rhomboid topology and is shown in *gray*. Catalytically active members are shown with a stronger colored background, whilst inactive members are fainter. Named subgroups are shown with respective labels and arcs along the tree with secretase subgroup classifications from the study by Lemberg *et al.* (10). *Yellow stars* denote published structures (human iRhom2 (also known as RHBDF2) (56, 57), human Derlin-1 (60), yeast Der1 (61), hiGlpG (58), and ecGlpG (59, 84)). UniProt (159) sequences used are available in Table 1. Neighbour-joining function was performed by the CLUSTALW function with the MEGA11 software package (version 11.0.13).

**Table 1:** Rhomboid members characterized in the literatures, and displayed in Figure 1, with alternate names or synonyms in the literatures given in column 2 (where appropriate)

Rhomboid	Alternative names	Species	Substrate/interaction partner (∗)	Reported function	UniProt code
GlpG	—	*Escherichia coli*	—	—	P09391
GlpG	—	*Haemophilus influenzae*	—	—	P44783
GlpG	—	*Shigella sonnei*	HybA, HybO∗, FdoH∗, YtjC∗, YqjD∗ (8)	Protein quality control (8)	Q3YWA4
Rhom7	—	*Shigella sonnei*	FdnH∗, HybA∗, HybO∗ (8)	Protein quality control (8)	Q3Z4D6
AarA	—	*Providencia stuartii*	TatA (15)	Quorum sensing (15)	P46116
YqgP	GluP	*Bacillus subtilis*	MgtE (106)	Protein quality control (106)	P54493
RhoX	—	*Brucella abortus*	—	Reported hypoxia adaptation (120)	Q2YQG9
RssP	—	*Vibrio cholerae*	VesB (121)	Protein localization/secretion (122)	Q9KQL7
PA3086	—	*Pseudomonas aeruginosa*	—	—	Q9HZC2
CpRom1	ROM1	*Cryptosporidium parvum*	—	Possible role as part of extracellular vesicles (123)	M9QCV8
BfROM1	—	*Bacteroides fragilis*	—	—	A0A016AI53
PaROM	—	*Psuedomonus aeriuginosa*	—	—	Q9HZC2
SpROM	—	*Streptococcus pneumoniae*	—	—	A0A6G2DA21
BfROM2	—	*Bacteroides fragilis*	—	—	A0A016ADA0
RbdB	—	*Aspergillus fumigatus*	—	Hypoxia adaptation, pathogenesis (124)	Q4WLP9
AqROM	—	*Aquifex aeolicus*	—	—	O67346
Rho1	—	*Haloferax volcanii*	—	—	A0A6C0UTM7
Rho2	RhoII	*Haloferax volcanii*	—	Bacterial motility, glycosylation regulation (125, 126)	A0A6C0UPU8
Rhomboid 1	Rv1337	*Mycobacterium tuberculosis*	—		P9WM21
Rhomboid 2	Rv0110	*Mycobacterium tuberculosis*	—	Homologous activity to AarA (*Providencia stuartii*) (33)	E2IBS4
Rhomboid 1	MSMEG_5036	*Mycobacterium smegmatis*	—		A0QT63
Rhomboid 2	MSMEG_4904	*Mycobacterium smegmatis*	—	Biofilm formation, antimicrobial protection (33)	E2IBT5
Rho-1	rho, DMRHO, DmRho1, Rho1, DMRHOa, DMRHOb, DRORHO, iks, RHO, veinlet, Ve	*Drosophila melanogaster*	Spitz, Keren (3)	Epidermal growth factor receptor (EGFR) signaling (13)	Q540V7
Rho-2	B-Rho, Brho, Stet	*Drosophila melanogaster*	Gurken (13)	EGFR signaling (13)	Q9W0F1
Rho-3	Rho-related, Rho3, Ru	*Drosophila melanogaster*	Spitz, Keren (13)	EGFR signaling (13)	Q9W0F8
Rho-4	DmRho4, Rho4	*Drosophila melanogaster*	Spitz, Keren (13)	EGFR signaling (13)	Q9VYW6
Rho-7	PARL, R7	*Drosophila melanogaster*	DmOpa1-like, DmPINK1 (13)	Mitochondrial fusion, mitophagy (13)	A1Z8R8
RHBDL1	RHBL	*Homo sapiens*	—	—	O75783
RHBDL2	RHBL2	*Homo sapiens*	IL-11R, BCAM, Spint-1, DDR1, CLCP1, KIRREL, IL6R (127, 128)	Immune signaling, epithelial homeostasis (127, 128)	Q9NX52
RHBDL3	RHBL3, RHDBL4, VRHO	*Homo sapiens*	—	Neuronal development/protection (129)	P58872
RHBDL4	RHBL4, RHBDD1, RRP4	*Homo sapiens*	DDR1, KIRREL, CLCP1, APP, Erlin1, Erlin2, p97, IRS4, PGAM5, GORS2, BIK, OST,pTα, Ubiquitin∗, BiP (54, 67, 105, 128, 130, 131, 132, 133)	Removal of C-terminal endopalsmic reticulum (ER)–retention motifs, membrane protein quality control, Recognition of ubiquitinated membrane proteins in ER membrane and ERAD (132)	Q8TEB9
RHBDD3	—	*Homo sapiens*	DAP12 (55)	Regulates natural killer cells (55)	Q9Y3P4
PARL	PSARL	*Homo sapiens*	PINK1, PGAM5, STARD7, HTRA2, OPA1 (134, 135)	Mitochondrial fusion, mitophagy, mitochondrial protein partitioning (17, 134, 135)	Q9H300
ROM1	Rhomboid-related protein 1	*Caenorhabditis elegans*	LIN-3 EGF (136)	EGFR signaling (136)	Q19821
Pcp1	RBD1, MDM37, UGO2	*Saccharomyces cerevisiae*	Immature cytochrome oxidase *c* (iCccp1), Mgm1 (137, 138)	Mitochondrial respiration, protein maturation, mitochondrial morphology (137, 138)	P53259
Rbd2	Rhomboid protein 2	*Saccharomyces cerevisiae*	—	Mitochondrial membrane remodeling (139)	Q12270
Rbd2	Golgi rhomboid protease, rhomboid protein 2	*Schizosaccharomyces pombe*	Cdc48∗ (95)	Hypoxia adaptation (95)	O74926
RBL1	At2g29050	*Arabidopsis thaliana*	—	—	Q0WQX7
RBL2	At1g63120	*Arabidopsis thaliana*	—	—	Q9CAN1
RBL8	KOMPEITO, At1g77860	*Arabidopsis thaliana*	—	Pollen exine wall morphology (140)	F4I8K2
RBL9	RBL11, At5g25752	*Arabidopsis thaliana*	—	—	Q84MB5
RBL10	RBL8, At1g25290	*Arabidopsis thaliana*	ACP4∗, CTI1∗, PAP8/FBN6, ZKT/MET1∗, PGK1∗, SBPase∗, STR10∗, PGDH1/EDA9∗ (141)	Jasmonic acid biosynthesis, lipid metabolism (chloroplast) (141), flower development (140)	F4ICF4
RBL12	AtPARL, At1g18600	*Arabidopsis thaliana*	—	—	Q9FZ81
RBL16	At1g74130	*Arabidopsis thaliana*	—	—	Q84WG3
PfROM1	—	*Plasmodium falciparum*	AMA1, Rh1, Rh2b, Rh4 (28)	Parasitic infection (28)	A8IWX2
PfROM4	—	*Plasmodium falciparum*	Rh adhesins, EBL adhesins (EBA-175), BAEBL (28, 142)	Parasitic infection, erythrocyte invasion (142)	A0A024VHB8
PbROM1	—	*Plasmodium berghei*	—	Parasitic infection, development (143)	A0A0Y9WRY1
PbROM3	—	*Plasmodium berghei*	—	Sporozoite development (144)	A0A0Y9VJS1
PbROM4	—	*Plasmodium berghei*	—	—	A0A0Y9XL14
PbROM6	—	*Plasmodium berghei*	—	—	A0A0Y9ZYX4
PbROM7	—	*Plasmodium berghei*	—	Asexual development (144)	A0A0Y9XZN3
PbROM8	—	*Plasmodium berghei*	—	Asexual development (144)	A0A1C6WZ38
TgROM1	—	*Toxoplasma gondii*	—	—	Q695U0
TgROM2	—	*Toxoplasma gondii*	—	—	Q695T9
TgROM3	—	*Toxoplasma gondii*	—	Asexual development (144)	Q6IUY1
TgROM4	—	*Toxoplasma gondii*	AMA1 (145)	Asexual development (144), parasite invasion (29)	Q695T8
TgROM5	—	*Toxoplasma gondii*	MIC2, MIC6, MIC12 (146)	Parasite invasion (29)	Q6GV23
TgROM6	—	*Toxoplasma gondii*	—	Asexual development (144), possible PARL homolog (10)	Q2PP52
TvROM1	—	*Trichomonas vaginalis*	TVAG_166850, TVAG_280090, EBA-175 (32)	Cellular attachment, cytolysis (32)	A2F737
TvROM2	—	*Trichomonas vaginalis*	—	—	A2DT69
TvROM3	—	*Trichomonas vaginalis*	—	—	A2DAB8
TvROM4	—	*Trichomonas vaginalis*	—	—	A2E4V6
EhROM1	—	*Entamoeba histolytica*	Gal/GalNAc lectins (147)	Parasite adhesion, motility, phagocytosis (148, 149)	M3USQ1
EtRom3	Rhomboid-like protein	*Eimeria tenella*	MIC3 (150)	—	U6L292
Derlin-1	DER1, Der1, hDER1	*Homo sapiens*	VCP/p97∗, Surf4∗, Derlin-2∗, SNX2∗, Snx1∗, SNx6∗, COX-2∗ (60, 117, 151)	Protein quality control, ERAD (6, 18, 152)	Q9BUN8
Derlin-2	DERtrin-2, DER2, F-LAN-1, FLANA, Derl2	*Homo sapiens*	BAG6∗, COX-2∗, Surf4∗, SNX2∗, Snx1∗, SNx6∗ (49, 117)	Protein quality control, ERAD (6, 18, 152)	Q9GZP9
Derlin-3	DER3, LLN2, Derl3	*Homo sapiens*	SLC2A1 (Glut1)∗ (153)	Protein quality control, ERAD (6, 18, 152)	Q96Q80
Derlin-1	Der1, Derl1	*Mus musculus*	Der2∗, VIMP∗, p97∗, SNX2∗ (19, 151)	Protein quality control, ERAD (6, 18, 152)	Q99J56
Derlin-2	Der2, Derl2, Flana	*Mus musculus*	Der1∗, VIMP∗, p97∗, SEL1L∗, HRD1∗ (19)	Protein quality control, ERAD (6, 18, 152)	Q8BNI4
Derlin-3	Der2, Izp6, DERtrin-3, Derl3	*Mus musculus*	Herp∗, SE1L1∗, HRD1∗ (19)	Protein quality control, ERAD (6, 18, 152)	Q9D8K3
Derlin-1	Der-1	*Drosophila melanogaster*	—	Protein quality control, ERAD (6, 18, 152)	Q9VQ57
Derlin-2	dDer-2, DER1	*Drosophila melanogaster*	—	Protein quality control, ERAD (6, 18, 152)	Q9VEU2
Derlin-1	Der-1, CUP-2	*Caenorhabditis elegans*	SNX-1∗, SEL-1∗, SEL-11∗ (46, 151, 154)	Protein quality control, ERAD (6, 18, 152)	Q93561
Derlin-2	DER1-like protein 2, cDerlin-2	*Caenorhabditis elegans*	—	Protein quality control, ERAD (6, 18, 152)	Q21997
Der1p	Der1, Der-1, Derlin-1	*Saccharomyces cerevisiae*	—	Protein quality control, ERAD (6, 18, 152)	P38307
Dfm1	DER1-like family member	*Saccharomyces cerevisiae*	Cdc48∗, Hmg2∗, Orm2∗ (20)	Protein quality control (20, 152, 155)	Q12743
Der1-like	—	*Schizosaccharomyces pombe*	—	—	O94458
Dsc2	Ucp14	*Schizosaccharomyces pombe*	Dsc E3 ligase components, Cdc48∗ (156)	Hypoxia adaptation (156)	Q9UTK7
Derlin	—	*Entamoeba histolytica*	—	Assumed protein quality control through ERAD (6, 18, 152)	M3TY80
RHBDF1	iRhom1, DIST1	*Homo sapiens*	Auxilin-2∗ (108)	—	Q96CC6
RHBDF2	RHBDL5, RHBDF6, iRhom2	*Homo sapiens*	K16∗ (50)	Neurological development, membrane protein quality control, cancer metastasis (47)	Q6PJF5
RHBDD2	RHBDL7	*Homo sapiens*	—	Protein quality control, ERAD, transforming growth factor α signaling (35, 48)	Q6NTF9
TMEM115	Placental protein 6, protein PL6	*Homo sapiens*	—	—	Q12893
UBAC2	PHGDHL1	*Homo sapiens*	UBXD8 (157)	Energy homeostasis (157)	Q8NBM4
RHBDF1	iRhom1, Dist1, Kiaa4242	*Mus musculus*	—	Neurological development, membrane protein quality control (5, 18)	Q6PIX5
RHBDF2	RHBDL6, Rhor, iRhom2	*Mus musculus*	AREG∗, FRMD8∗ (5, 109)	Neurological development, membrane protein quality control (5, 18)	Q80WQ6
Rho-5	iRhom	*Drosophila melanogaster*	—	—	Q76NQ1
ROM2	iRhom2, C48B4.2	*Caenorhabditis elegans*	—	—	P34356

Native substrates or interaction partners only are displayed for visual ease, and the thick horizontal border delineates active protease (above), and inactive protease family members (below). Interaction partners (*i.e.*, those that are known to physically associate with respective rhomboids but are not cleaved or known to be cleaved) are denoted with a ∗. UniProt code is specifically listed as these were the sequences used for generation of Figure 1.

In this review, we aim to describe what is currently known about the rhomboid family, give an overview of the functional roles of both active and inactive proteases and their conserved structure, as well as highlight recent developments in the field with regard to high-resolution structural determination of pseudoproteases Derlin and iRhom2. We also aim to highlight the relationship between rhomboid members and their native membrane environment, and what this relationship means for rhomboid function as a family. As this work spans the interaction of rhomboid family members with different proteins, leading to different functional outcomes, we make specific distinctions between a substrate (a protein that is proteolytically cleaved by an active rhomboid protease), interaction partner (a protein that is known to associate with either an active or inactive rhomboid protease but is not either cleaved or removed by the misfolded protein pathways such as endoplasmic reticulum (ER)–associated degradation (ERAD), and a client protein (a misfolded protein that is detected by either Derlin or iRhom members, which is then removed by a misfolded protein pathway or chaperoned to a folded state).

## The many roles of rhomboid family members

**Figure 2:**
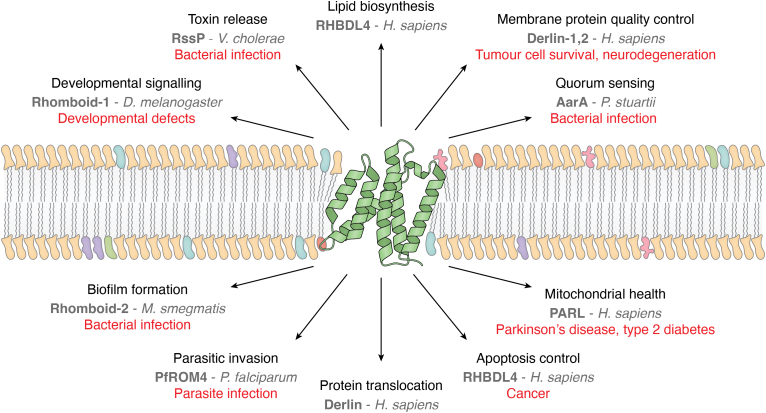
**Representative structure of rhomboid member in lipid bilayer, with a subset of known physiological roles of rhomboid family members (both active and inactive proteases) found in the literature**. Physiological roles are described in *black* and specific member examples given in dark *gray*. Corresponding disease states for dysfunction are shown in *red*.

The specific functions of rhomboids are difficult to generalize because of the presence of both active and inactive protease members and the sheer number of members in the superfamily. However, it can be said that, generally, the roles these members play are often highly conserved (Fig. 2) and are involved in protein–protein interactions, either resulting in protein proteolysis directly (active catalytic members), indirect modulation of other protein functions (iRhoms), or through chaperone or ERAD mechanisms (iRhoms and Derlins). For catalytically active proteases, some notable examples range from quorum sensing in bacteria (AarA in *Providencia stuartii*) (15), growth factor signaling in both *Drosophila* (Rhomboid-1 and EGF/Spitz signaling) (3) and Arabidopsis (Atg74130) (16), to metabolic health in humans (PARL) (17). Catalytically inactive proteases, otherwise known as pseudoproteases, perform functions primarily in protein quality control. These include the monitoring and regulation of the protein quality control in eukaryotes (Derlin directly in ERAD, and Derlin-3’s involvement in fine tuning the ERAD response) (18, 19), direct chaperone functions of misfolded membrane proteins (Derlin homolog Dfm1 in yeast) (20), to involvement in the secretion of tumor necrosis factor α as part of the inflammatory response (RHBDF2 (also known as iRhom2)) (21).

Bioinformatic study has shown that the rhomboid family is present across all domains of life, and conservation of both active and inactive protease members indicates that the last universal ancestor likely had two copies of an ancestral rhomboid (22). This is in line with the fundamental nature of most roles undertaken by both types of rhomboid members. The ability of distantly related rhomboid proteases to cleave exogenous substrates, rescue loss-of-function mutations or knockouts in some *in vivo* or *in vitro* experiments (9, 23, 24), aids in this argument. Similarly, it has been shown by Kandel *et al.* (20) that human Derlins (Derlin-1 and Derlin-2) were able to at least partially restore ERAD function in Dfm1 (a Derlin equivalent protein)-deficient cells in yeast. This exogenous substrate compatibility has been useful in the study of rhomboid proteases and has allowed for the characterization of how these members select substrates (25).

Despite lacking complete functional data for the majority of rhomboid members, there are several instances of rhomboids being involved in clinically relevant processes (Fig. 2). Most notably, the protozoan rhomboid proteases are well known to be heavily involved in parasitic invasion and life cycle and seem to be functionally highly conserved between genera (26, 27, 28, 29, 30, 31, 32). Bacterial proteases have been less well functionally described but shown to play key roles in quorum sensing, biofilm formation, and cell division (15, 33, 34). Outside parasitic and bacterial infections, human rhomboid proteases have been found to play a role in numerous cancers, such as RHBDL4 in breast cancer and colon cancer, most likely through an activation of Wnt or epidermal growth factor receptor signaling (35, 36, 37). PARL, the human mitochondrial rhomboid protease, is known to play a key role in the regulation of mitophagy and apoptosis (38, 39, 40). This naturally leads to PARL dysfunction being implicated in numerous disease states, such as metabolic (most notably type 2 diabetes) (41, 42) and neurodegenerative diseases (Parkinson’s and Alzheimer’s) (43, 44), as well as cancer (45). Similarly, pseudoproteases have been implicated in cancer (46, 47), with their dysfunction most likely being related to aberration of correct membrane protein quality control (48). This has been reviewed recently and more comprehensively by Burzenski *et al.* (5). Other examples include Derlin-2 being implicated in aiding chemotherapy resistance through increasing the half-life of procancer protein BAG6 (49), RHBDD2 being shown to be upregulated in breast cancer (48), and RHBDF2 (otherwise known as iRhom2) being shown to directly affect keratin (K16) levels, which can lead to cancer (50).

Despite their fundamental nature and clinical significance, this family of proteins is still understudied. Whilst significant progress has been made in characterizing the function of specific family members since *Drosophila*’s Rhomboid-1 in 2001, there is still a lack of structural and comparative biochemical studies to understand the various roles of members. This is partly because of the complex and transient interactome these rhomboids exist within, especially for inactive rhomboid protease members. However, recent advances have begun to shine light on the role of these catalytically inactive but necessary proteins.

## The conserved structure of the rhomboid family

### Overall structure

**Figure 3:**
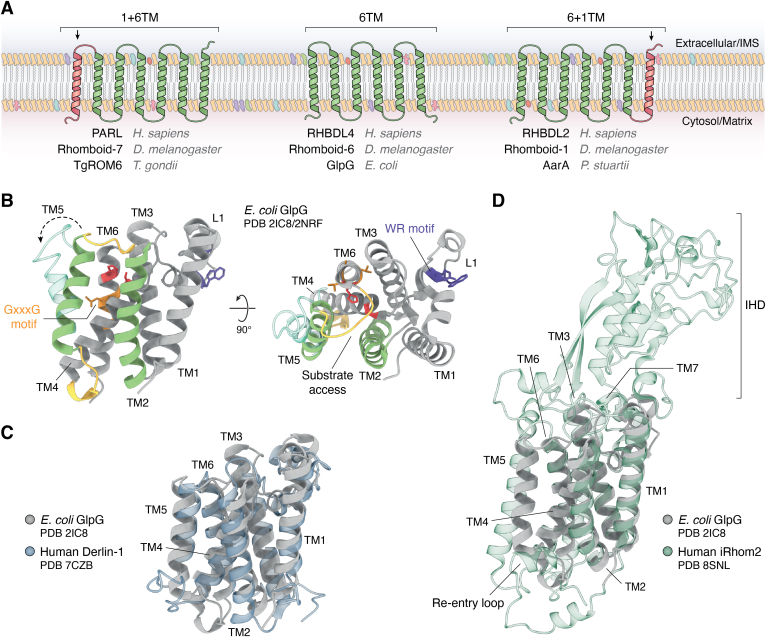
**General topology and common structural features of rhomboid superfamily members.***A*, representative topology models showing the transmembrane helix arrangement of rhomboid family members (1+6, 6, 6+1, *left to right*), with representative human, fly, and “other” family members shown below. *B*, PDB structure of *Escherichia coli* GlpG (PDB ID for *opaque cartoon*, showing “closed” structure: 2IC8, and PDB ID for *transparent overlay* of TM5 showing “open” conformation: 2NRF) with commonly conserved motifs shown in *orange* (GxxxG helix dimer motif), *purple* (WR motif), and *red* (Ser-His dyad), where appropriate. Gating helices are shown in *green*, with dynamic loops connecting these in *yellow*. *C*, overlay of *E. coli* GlpG (PDB ID: 2IC8, *gray*) and human Derlin-1 (PDB ID: 7CZB, *blue-transparent*) to highlight common structural fold across domains of life. *D*, overlay of *E. coli* GlpG (PDB ID: 2IC8, *gray*) and human iRhom2 (PDB ID: 8SNL, *green-transparent*) to highlight common structural fold across domains of life; the large addition of the cytosolic iRhom homology domain is labeled IHD. PDB, Protein Data Bank.

As mentioned previously, the rhomboid superfamily is broadly defined by a common “rhomboid-like” fold, this being a compact bundle of six-TM α-helices. This fold, whilst variable in exact protein sequence, has proven useful to identify potential members in bioinformatic searches (22). However, the majority of these potential members are yet to be verified experimentally. Additional helices on either the N- (1+6) or C termini (6+1) may be present (Figs. 1 and 3*A*), with the former primarily belonging to members functioning within eukaryotic organelles, such as plastids and mitochondria, and the latter found primarily in the eukaryotic secretory pathway (11, 51). Soluble domains have been observed and are thought to provide supplementary functionality, such as rhomboid-4’s calcium-binding EF-hands motifs (52), GlpG’s N-terminal domain, modifying the orientation of the protein in the membrane (53), as well as RHBDL4’s C-terminal extension containing an ubiquitin-binding motif (54). These functional extensions are not limited to active proteases. The inactive protease members RHBDD3, Dsc2, and UBAC2 also contain a ubiquitin binding–associated domain (14, 55), and the cytosolic N terminus of pseudoprotease RHBDF2 (iRhom2) has been shown to be essential for regulation of inflammation signaling in mice models (5, 21), as well as the regulation of ADAM17 proteolytic activity in humans (56, 57). But as always, more research into the supplementary effect of these extrarhomboid domains is required for individual family members.

Experimentally derived structures have as yet been limited, with only a few members of the family having structures determined. The bacterial GlpG (two homologs, *E. coli* and *Haemophilus influenzae* (58, 59)) of the active proteases and the pseudoprotease human Derlin-1 and yeast homolog Dfm-1 as part of the ERAD complex (60, 61, 62) as well as the recent addition of human iRhom2 in complex with ADAM17 metalloprotease (56, 57). All these structures highlight the bioinformatically predicted conserved structural fold of the TM region with six α-helices in a tight bundle (Fig. 3, *B*–*D*); however, some additional domains and rearrangements can be observed, especially for the iRhom2 structure; the additional helix TM7, the iRhom homology domain (Fig. 3*D*, IHD), and the small additional re-entry loop between TM1 and 2.

### Conserved sequence motifs

Some conserved and functionally important sequence motifs are found in the majority of members, originally predicted through bioinformatics, and confirmed with mutational studies (Fig. 3*B*). Most notable are the structurally important GxxxG helix dimerization motif on TM4 (3) and the WR motif (tryptophan–arginine) in loop L1 (3).

The helix dimerization motif GxxxG has been shown to be key for rhomboid structural stability (63, 64), enabling tight packing of TMs 4 and 6 (22, 65). Abrogation of this sequence leads to a loss of function in both the active proteases (66, 67) and inactive Derlins (67, 68). However, more work is needed to explore functional conservation of this sequence for Derlins, as it has been shown that for yeast Derlin Der1, the “GxxxG” sequence is instead NxxxR with seemingly no effect on function (6), and the same article showed that the GxxxG–NxxxR sequence alone was not sufficient for substrate binding but the WR motif was.

The WR motif in loop L1 is present in approximately 80% of rhomboid members (strictly conserved in the “A” class secretases but sequence conservation of xR for “B” class secretases as defined by Lemberg *et al.*) (10, 69) and is thought to play a role in structural stability and positioning within the membrane. It is thought that loop L1 in general is a key driver for the rhomboid orientation within the bilayer and the subsequent lipid thinning (69, 70) that appears to be a hallmark of rhomboid family member function (53). It is known that loop L1 is partially embedded within the membrane (71), with the WR member tryptophan playing a role in the correct orientation of the protein in the membrane through hydrophobic shielding (72). It has also been shown that arginine interacts heavily with the L1 loop, generating a rigid structure and stabilizing the loop at the membrane interface (71). Mutational studies of these residues have shown that substitution of the arginine in this pair abolishes catalytic activity in both RHBDL2 (3, 73) and GlpG (66) in detergent-based assays, with the same studies showing mutation of the tryptophan residue only reducing catalytic activity, similar to other lipid-associating residues in loop L1 for GlpG. Interestingly, alanine substitution of the arginine in rhomboid protease YqgP only reduced, not abolished, activity in detergent-based assays (74), and activity assays on GlpG *in vivo* showed that cysteine substitution for both tryptophan and arginine only reduced activity (although significantly so) (72). For noncatalytically active rhomboids, the WR motif is also necessary for function. Yeast Derlin Dfm1 requires the WR motif for recognition of membrane substrates (6), although it is not sufficient for retrotranslocation. However, the same exception for GxxxG is present here for WR, where in yeast Der1, the “WR” pair is instead a “GR” with seemingly no functional loss (6).

For active proteases, there are two additional conserved sequences: GxSx for the catalytic serine and A/GH for the corresponding catalytic histidine. This sequence conservation is common for catalytic residues, as the correct chemical environment is needed. For example, the trypsin subfamily of serine peptidases contains a conserved sequence of DSGGP and TAAHC for serine and histidine, respectively (75).

### The role of oligomerization

The presence of the helix dimerization motif in particular has led to discussions over whether rhomboid members are functional *in vivo* as monomers, or in oligomeric states, similar to other membrane protein families, such as G protein–coupled receptors (76, 77). However, no evidence has been presented that shows the GxxxG motif in rhomboids is anything but an intraprotein structural stabilizer. Some studies have shown that there is a proclivity for some rhomboid members to oligomerize in detergent micelles (78, 79) and that this oligomerization increases the rate of substrate cleavage (80). However, studies performed under more physiological conditions (*i.e*., *in vivo* or within lipid bilayers) show no significant dimerization for active protease members thus far (52, 81). Work therefore needs to be done to differentiate between members that can oligomerize (with or without biological function) and the general statement that rhomboid family members oligomerize as a familial trait. Having said this, it is known that inactive rhomboid proteases, such as the members of the Derlin subfamily involved in ERAD, form both homo- and hetero-oligomers *in vivo* (82), with some evidence that the component mixture of this heterodimerization plays a functional role (19). Recent structural work using detergent micelles has shown that human Derlin-1 forms tetramers to form a membrane-spanning channel as part of ERAD retrotranslocation (60), with authors hypothesizing that Derlin monomers are the substrate-recognizing form, whilst the tetrameric form is necessary for ERAD retrotranslocation. However, this does not explain *in vivo* work showing that the majority of human Derlin-1 exists as a homodimer, seemingly in an inactive state (83).

### Overall topology and active site accessibility

**Figure 4:**
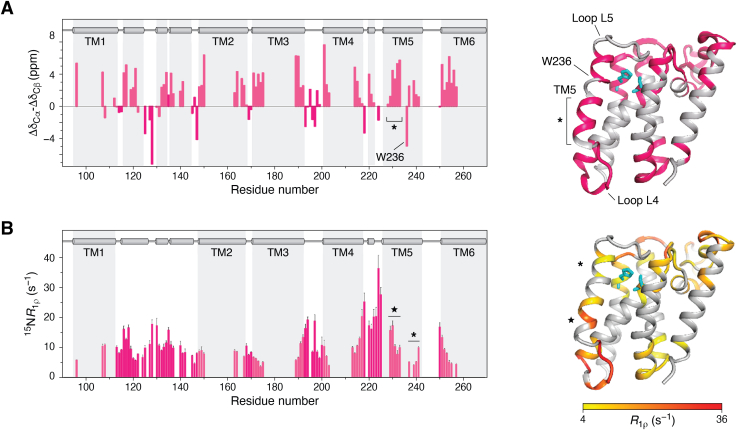
**Conformational dynamics of Escherichia coli's GlpG as measured by solid-state NMR.***A*, secondary chemical shift analysis, revealing a kink at W236. *Left,* assigned residues in *magenta* (*right*). TM5 is the only solvent-accessible TM, which leads to H/D exchange and thus NMR-observable H/N signals. The other TMs therefore remain “*gray.*” *B*, relaxation analysis (R_1ρ_ as a function of residue number), showing dynamics dominated by motion on the microsecond timescale (where higher numbers indicate faster dynamics). The C terminus *(upper*) and N terminus (*lower*) are marked with an *asterisk* and a *star*, respectively. Figure adapted and reprinted with permission from Shi *et al.* (86). Copyright 2019 American Chemical Society. H/D, hydrogen/deuterium; TM, transmembrane.

Much debate has occurred around the topology and (where relevant) active site position in relation to potential substrate interaction sites. Whilst it is clear from the structures of both *E. coli*’s and *H. influenzae*’s GlpG that the catalytically active dyad is located within the hydrophobic core of the bilayer, how the compact α-helical bundle of the rhomboid fold accommodates substrate interaction remained a challenge from early structures. The first structures of a rhomboid family member were published in 2006, both of which depicted *E. coli*’s active protease GlpG (with the cytosolic N-terminal domain truncated), and later in 2007, a crystal structure was published of a homolog of GlpG in *H. influenzae* (58, 59, 84). The structure presented by Wang *et al.* (59) showed a tight α-helical bundle, as predicted, and proposed that access to the active core was controlled by loop 1, which in their structure showed an amphipathic nature. This was supported by the conserved nature of the WR motif in this region and its importance for activity. This loop architecture was similarly observed by Ben-Shem *et al.* (85) in a publication of GlpG just months later. However, the crystal structure presented by Wu *et al.* (84) contained an asymmetric crystal unit, one of which showed a tight α-helical bundle as previously stated, and one possessing a partial TM5 helix kink. This was predicted by Wu *et al.* to represent a substrate gate between TMs 2 and 5, with the tight bundle being representative of the “closed” state, and the kinked TM5 being representative of the “open” or “partially open” state. A later crystal structure of *H. influenzae*’s GlpG showed weak electron density for TM5, which the authors predicted indicated conformational dynamics of this region. However, these three initial crystal structures were determined in detergent-micelle conditions. A later crystal structure within a lipid environment (bicelles) was published by Vinothkumar *et al.* (71) and minimized the importance of the TM5 helix in substrate gating, instead favoring loop 5 dynamics. Eventually however, solid-state NMR (ssNMR) of GlpG within *E. coli* liposomes by our group confirmed that the TM5 helix is indeed dynamic, and that this conformational exchange readily occurs in a native-like environment (86, 87). Our data revealed the presence of a kink at W236 for GlpG in liposomes (Fig. 4*A*) and relaxation data—dominated by motion on the microsecond range—showed that TM5 is a dynamic hotspot (Fig. 4*B*), as well as the adjacent loop L4. On the other end of TM5, loop L5 appears even more dynamic so that it cannot be detected in regular (dipolar coupling–based) ssNMR experiments (86). Surprisingly, hydrogen/deuterium data suggested TM5 to be solvent exposed, at least when solubilized in detergent, whereas all other TMs are protected (Fig. 4*A, right*). Finally, comprehensive mutational analysis of residues thought to be lining the proposed TM2 and TM5 gates by Baker *et al.* (66) showed that mutation of proposed gating residues between TMs 2 and 5 to either alanines (increased dynamics) or cysteines (crosslinking to reduce dynamics) corresponded to a proportional increase and decrease in substrate processing, respectively. This was followed by work from Moin *et al.* (88), which showed mutation of these residues can affect the cleavage position of the protease, indicating that site specificity is partially dependent on gate opening. Our group recently showed that mutation of these “gating residues” along TM2 and 5 indeed corresponded to a proportional increase (more flexible) and decrease (less flexible) in substrate-processing rates in native-like membranes. We also showed, through ssNMR, that this effect is due to increased TM5 dynamics, as well as a decrease in structural stability of GlpG as a whole when the residues are mutated from their native large hydrophobic groups, and not crosslinked (89). It is worth noting that all structural studies of GlpG have, until recently, been performed only on a truncated version of the protease, known as GlpGΔN (because of the missing cytosolic N-terminal region). This was initially necessary for crystal formation for crystallography, and to minimize signal overlap in ssNMR spectra; however, new sensitivity-enhancing techniques developed by our laboratory have recently resulted in the first structural information for full-length (wildtype) GlpG (90).

Pseudoprotease characterization and structure have historically been neglected; however, recent structures have shown commonality between proteolytic active and inactive members in overall fold. Two cryo-EM structures of human Der1 have been produced in the past 5 years by Rao *et al.* (60, 62), as well as Der1 in yeast by Wu *et al.* (61), with these structures showing the common tight α-helical bundle arrangement originally observed in GlpG. Similarly, two structures of human iRhom2 have been published in complex with client protein ADAM17, again showing the same overall tight bundle as active protease GlpG. Mutational work by both groups also showed that removal of the TM association between iRhom2 and ADAM17 removed ADAM17 activity, indicating that—once again—the rhomboid family’s function is primarily transmitted through protein–protein interaction through the TM region. However, unlike the active proteases, this interaction point is mediated through TM1. Interestingly, the structures from Rao *et al.* also indicate that the interaction with client proteins is similarly distinct from the substrate gate in active proteases. The tetrameric human Derlin-1 structure showed that tetramerization, thought to be necessary for ERAD retrotranslocation, confines the TM2 and 5 helices to the inner side of the formed channel. They instead propose that TMs 1 and 3 form a lateral gate that may serve as an entrance for client passage to the channel prior to ERAD translocation (60). This is further supported by the dynamics of the tetramer being observed to show large conformational dynamics of the TM1, TM2, and loop 4 of Derlin-1, when complexed with retrotranslocation machinery, p97 (62).

## How do rhomboid family members find their substrates or interaction partners?

Biological membranes are a hydrophobic sea, with protein sequences within this region evolving more for stability than specific binding motifs. In this environment, how do rhomboids find their interaction partners, client proteins, and substrates? This has been a question that both protease and pseudoprotease researchers have been at odds to answer (for a more wide-ranging review on intramembrane protease substrate recognition, we recommend the following review from Lemberg *et al.* (91)). Whilst no specific recognition sequence has been discovered generally for all rhomboid members, it does appear that—at least for proteases—there are some common features of known substrates.

### Active proteases: substrate motifs and extramembrane residues

**Figure 5:**
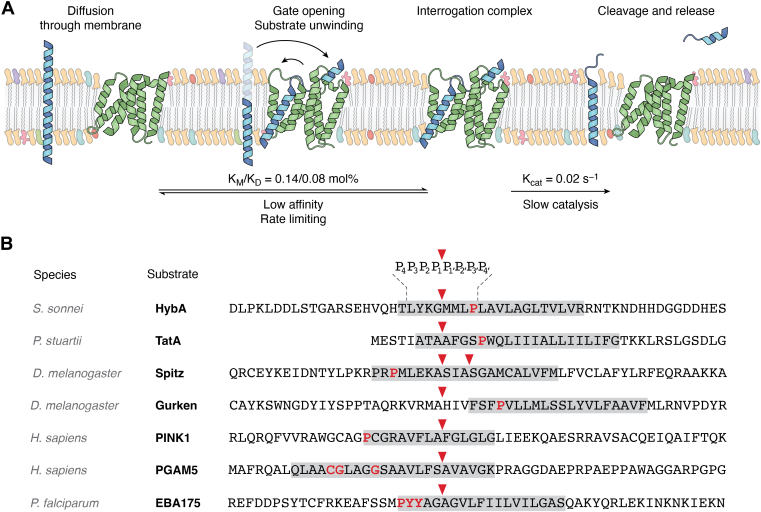
**Rhomboid protease-substrate interaction within the membrane and sequence-dependent substrate specificity.***A*, representative cartoon of interaction between active protease rhomboid family member and substrate in native-like membranes. The *top text* shows the likely steps between stages (from substrate recognition to cleavage), and the *bottom text* shows the relative kinetics of each step, as observed for *Escherichia coli*’s GlpG by Dickey *et al.* (94). *B*, sequences of commonly used and characterized substrates where the cleavage sequence is known. The *top row* shows format (species, substrate, and sequence), with commonly used residue monikers P_1–4_ and P′_1–4_ around the *redcleavage arrow*. *Aligned arrow* per substrate denotes cleavage site in the literature by either model protease GlpG (HybA, TatA, Spitz, and Gurken) or native protease (PARL: PINK1, PGAM5, PfROM4: EBA175); *secondary arrow* (Spitz) shows secondary cleavage site by DmRom4 known *in vivo* (52). *Gray boxes* denote predicted transmembrane domain by TOPCON (158), and *red* residues in *bold* denote predicted destabilizing prolines or known destabilizing or cleavage rate–affecting residues for EBA175 (PYY (92)) and PGAM5 (CGxxxG (96)).

The vast majority of substrates are single-pass, α-helical, TM proteins (Fig. 5*B*). This structural proclivity may indicate an evolutionary origin to rhomboid proteolysis: the preferential regulation of orphaned and unincorporated membrane proteins of larger complexes (8).

Strisovsky *et al.* (25) showed that, for GlpG, there is some degree of selection for specific amino acids around the cleavage location. They observed a preference for a small residue at P1 (for nomenclature, please see Fig. 5*B*), whilst hydrophobic and preferably large residues are present for P4 and P2′ positions. Gandhi *et al.* (92) similarly showed that different substrate preferences for rhomboids from *Plasmodium falciparum*, PfROM1 and 4, are due to a single residue on the substrate at the P4 position. PfROM4 was able to cleave all but isoleucine- and valine-containing substrates at this position (the so-called “beta-branched” residues), whilst PfROM1 has reciprocal specificity. Notably, whilst the cleavage site is often just within or at the membrane interface, the sequences determining specificity are predicted to be at or above the membrane interface (Fig. 5*B*), so are not subservient to the same evolutionary pressure of the hydrophobic membrane environment. Cho *et al.* (93) showed that soluble peptide sequences representing the P1–P6 residues of a substrate were sufficient to develop water-soluble peptide aldehyde inhibitors to GlpG in the micromolar range. The best sequence was based on the P1–P6 residues of a known rhomboid substrate (RKVRMA, Gurken) that is also cleaved by GlpG, and subsequent structural work shows numerous interaction sites between this peptide and the extracellular loop L3 and active site residues. This short peptide inhibitor was then the basis for further time-resolved X-ray work, providing valuable structural details on the catalytic mechanism of GlpG, which is likely conserved among all active rhomboid proteases.

However, extramembrane substrate sequences alone are not deciding factors in substrate recognition. In the same inhibitor work, Cho *et al.* showed that inhibitor binding is not competitive with substrate cleavage, indicating that the rate-limiting step is not the catalytic mechanism but substrate recognition (Fig. 5*A*). This confirmed earlier work by Baker *et al.* (66), which showed that the conformational dynamics of the TM5 helix is the rate-limiting step in catalysis. In addition, kinetic values obtained by Dickey *et al.* showed that substrate recognition and gating in numerous rhomboid proteases was rate limiting, and that all rhomboid proteases were surprisingly inefficient in terms of *K*_*M*_ and resulting *K*_cat_ (94). They also showed that extramembrane sequences do not affect binding affinity but do affect the rate of catalysis. Taken together, this shows that rhomboid substrate interrogation within the membrane is the primary driver of selection, rather than the specific sequence of the extramembranous sequence within substrates. Some evidence has shown that this weak protein affinity allows for tuning of substrate selection and therefore modulating rhomboid protein function. For example, Rbd2 in *Schizosaccharomyces pombe* is only able to cleave SREBP (sterol regulatory element–binding protein) because of Cdc48 performing a recruiting and substrate-adapting role when SREBP is ubiquitinated (95).

The key to in-membrane specificity therefore appears to be driven by dynamics. Helix-breaking proline or glycine spans seem to determine substrate from nonsubstrate in mutational studies (8, 40, 88, 96, 97) (Fig. 5*B*), and helix unwinding is necessary for TM substrate processing by GlpG (98) (Fig. 5*A*). In addition to destabilizing residues, the hydrophobic thickness or gating dynamics experienced by protease and substrate have been shown to affect the specific scission site (88, 99), and one study on RHBDL4 indicates that differential cleavage by a rhomboid protease may be a homeostasis mechanism of membrane saturation (100). This shifting cleavage site by membrane properties is observed similarly in other membrane proteases, with one notable example being γ-secretase in the differential processing of β-amyloid precursor protein to produce the clinically relevant Aβ peptides (101).

Kinetics work by Foo *et al.* (102) has shown that the rate of substrate cleavage is dependent on the local thickness of the membrane bilayer or bicelle used, with GlpG cleaving substrates fastest in 1,2-dilauroyl-*sn*-glycero-3-phosphocholine lipids, and rapidly declining efficiency in thicker bilayers. Interestingly, 1,2-dilauroyl-*sn*-glycero-3-phosphocholine bilayers are approximately 31 to 33 Å thick, higher than the hydrophobic thickness of GlpG (∼25 Å by crystal structure determination (71)) and than the average membrane protein thickness of 29 Å (103). Engberg *et al.* (104) showed that GlpG is able to compress native-composition bilayers to between 24 and 26 Å and confirmed this was optimal for cleavage speed. It is unclear however whether bilayer thickness affects substrate recognition and dynamics specifically or simply the conformational equilibrium of the TM5 gating helix corresponding to a change in the rate of substrate cleavage (89). To complicate matters further, a recent proteomics-led search by Tang *et al.* (105) identified 43 potential RHBDL4 substrates, one of which was a soluble protein residing in the ER, BiP, with RHBDL4 acting as a noncanonical secretase. They went on to show that this cleavage occurs *in vivo* not only to BiP but to several other ER-resident soluble proteins, and that knockout of RHBDL4 stopped this proteolysis from occurring. It is unknown what determines a soluble substrate of RHBDL4, nor whether this type of cleavage is common among other rhomboid proteases.

### Regulation of substrate recognition or cleavage

Substrate recognition is only one aspect of protease activity, and biological processes require a degree of modulation to ensure a fine-tuned response dependent on different states or inputs. Rhomboid proteases are no different in this manner, and whilst the previous subsections have focused on selection of substrates from nonsubstrates, it is necessary to discuss the (somewhat limited) known examples of differential processing or substrate selection by rhomboid proteases depending on variable conditions. One example is of the selective cleavage of “orphan” substrates (*i.e.*, substrates that are not currently performing a function in a larger complex), such as *Shigella sonnei*’s GlpG and Rhom7 selective cleavage of HybA, FdoH, and FdnH only when without their respective complex partners (8). The more common regulation through sensing of a chemical concentration has also been discovered. One being the regulation of magnesium concentration, where high intracellular magnesium concentration leads to cleavage of magnesium transporter MgtE by YqgP in *Bacillus subtilis* (106), and another being the regulation of lipogenesis by RHBDL4, where higher relative levels of fatty acid saturation lead to preferential processing of the SREB-1c (100). It is also worth noting that RHDBL4 also appears to differentially cleave the substrates SREB-1c and amyloid precursor protein, depending on this saturation level, which is covered in more detail in a later section. A final example of rhomboid activity modulation is how post-translational modifications affect the rate of substrate processing. Post-translational modifications, such as phosphorylation, are a classical method of activity regulation, and one example is known for rhomboids: the mitochondrial rhomboid protease PARL. Phosphorylation of PARL reduces the formation of so-called “β-cleavage,” which leads to the production of PARLΔ77, and increases the relative levels of PARLΔ53, which is catalytically more active. The importance and specific methods of this regulation are still debated in the literature, and we point interested readers to a recent review by Lysyk *et al.* (107). As always, further work is required to develop a better understanding of not only substrate recognition by rhomboid proteases but also how this process is fine-tuned by outside factors.

### Inactive proteases: iRhoms and Derlins

Work on identifying the relationship between inactive proteases, their interaction partners, and client proteins has lagged behind active protease research, primarily due to difficulties in capturing these transient interactions in both *in vivo* and *in vitro* studies. Bioinformatics work on disease states, mutational studies, and recent structural models has begun to shed light on how these proteins distinguish client proteins and interaction partners from the sea of integral membrane proteins, and several interaction partners are now known (Table 1), particularly for non-ERAD system participants such as the iRhoms. iRhom1 has been shown to directly interact with pro-transforming growth factor α to induce its maturation to the secreting factor (37), as well as interacting with soluble auxilin-2 as part of the clatherin-coated vesicle transport pathway (108). iRhom1 has also been shown to interact with amphiregulin, and it is known that iRhom2 directly interacts with amphiregulin involved in wound healing (5, 109). How these interactions are mediated remains understudied but may involve the regulation of the activity of proteases, such as the metalloprotease ADAM17, where recent structural and mutational work shows four “interface” sites, three of which are cytosolic and involve the conserved additional iRhom homology domain (56, 57). Mutations of these interfaces only modified ADAM17 catalytic activity, with interface 3 (as named by Lu *et al.* (56), named as site 2 by Maciag *et al.* (57)) showing a significantly larger change than the other interface sites, although Lu *et al.* showed this as an increase in shedding activity (mutation of iRhom2’s D475), and Maciag *et al.* observed a decrease in activity (mutation of ADAM17’s residues R625, R626, and K628). However, the most interesting interface site for this review is within the transmembrane region, at interface 1. Mutation of these residues (iRhom’s I386 or ADAM17’s V673, V676, or L677) was shown by both groups to abolish ADAM17 activity, significantly reducing ADAM17 and iRhom2’s coprecipitation and ADAM17 maturation from the ER to the plasma membrane.

Similarly, mutational and coimmunoprecipitation studies have shown that Derlin members require TM association to identify misfolded proteins as clients (6). It has been shown that Dfm1 in yeast recognizes misfolded proteins through the conserved WR and GxxxG motifs (20), and that Dfm1 and human Derlin-1 possess the same lipid-thinning capabilities as observed in active protease members (6). This further implicates a “dynamics first” element in substrate recognition across the rhomboid family, grounded in localized membrane thinning. In addition, pseudoproteases must not only recognize client proteins but also form multimeric complexes with other interaction partners necessary for misfolded protein removal from the membrane. Structural work for Derlins shows the homo-oligomerization, and recruitment of ERAD machinery proteins is primarily performed through cytosolic-based interactions and the evolution of specific domains, such as the SHP box on Dfm1 or Derlin-1 (in yeast and humans, respectively), which is able to specifically recruit Cdc48, an ATPase known to enable retrotranslocation (VCP/p97 in humans) (60, 62). However, further work is required to fully explain the recruitment and homo-oligomerization of Derlin members as part of the ERAD machinery.

## Rhomboids within their lipid environment

Generally, the effect that the lipid environment of the membrane has on the evolution of membrane protein structure and function is difficult to overstate. In addition, the nature of an intramembrane protease requires several further solutions to the hydrophobic environment over less catalytically functional membrane proteins, most notably the access of water for catalysis, and substrate access. The research surrounding these hurdles was discussed in detail in the previous sections, and this section focuses on the specific interactions rhomboid proteins have with respect to the lipid membrane.

Initial *in vitro* studies for both active and inactive proteases showed that the highly conserved WR motif was necessary for the function of rhomboid family members, and it was proposed that this was due to a critical structural role and mediates protein–lipid interactions (12, 20, 70, 74, 110). Similarly, studies have highlighted that rhomboid proteases (namely *E. coli*’s GlpG) showed increased activity in detergent micelles over more native-like systems (74, 94, 111, 112), and that the activity in lipid bilayers was enhanced at a smaller hydrophobic thickness than expected for native *E. coli* membranes (102). In fact, the first structures of GlpG showed varied lipid thickness around the protein and were proposed to indicate a level of lipid remodeling, particularly around TM2 and 5 (“the gating helices”) (58, 59, 71). This remodeling was proposed to play a role in substrate recognition, whereby a combination of destabilizing residues within the TM region of the substrate and the locally thinned membrane would enable secondary structure unwinding and sequence interrogation prior to cleavage (70, 98) (Fig. 5*A*). However, no convincing evidence existed as to why this thinning occurs and whether this remodeling ability was exclusive to *E. coli*’s GlpG.

A landmark article by Kreutzberger *et al.* (53) showed that this thinning appeared universal to rhomboid members and that this aids diffusion of the rhomboid proteins within the membrane. In addition, this was the first article to show that pseudoproteases (specifically iRhom2) also possess this lipid-thinning ability, indicating a potential mechanism of their conserved function, which was as-of-yet unexplained (74). This experimental article confirmed earlier all-atom simulation work by Bondar *et al.* in 2009, which demonstrated lipid thinning by *E. coli*’s GlpG (both wildtype as well as several mutants) and predicted its function in substrate unwinding for cleavage (69). In addition, a combination of structural and mutational work with Der-1 in *Saccharomyces cerevisiae* (sometimes referred to as Derlin-1 in the early literature) confirmed that this thinning was present in multicomponent complexes containing Der-1 and was necessary for its effect on the ERAD pathway, through a separate mechanism to the also necessary WR motif (61). Engberg *et al.* (104) then showed that the lipid thinning observed for GlpG was due to specific interactions with phosphatidylethanolamine (PE) lipid headgroups, and that GlpG tightly associates with PE and phosphatidylglycerol (PG) lipids even through detergent solubilization, which confirmed earlier simulations by Bondar (70). Engberg *et al.* (99) went on to show that the rate of cleavage by GlpG was affected in noncompressible (such as 100% phosphatidylcholine (PC)) membranes. Work by our group also showed this PE and PG retention, with both detergent and polymer (DIBMA)-solubilized GlpG. In addition, we showed that N-terminally truncated GlpG, commonly used in structural studies, has a different response to the introduction of PC lipids than the wildtype—namely, the introduction of an alternative cleavage position in our model substrate TatA (see Supplementary Figure 6 in the study by Sawczyc *et al.* (99)). This aligns with continuous-wave electron paramagnetic resonance work by Kreutzberger *et al.*, which showed the N-terminal–deficient GlpG oriented in the membrane differently than wildtype (53), and simulation work by Almeida-Hernandez *et al.*, which showed that the cytosolic N-terminal domain affects GlpG–lipid interactions (113), as well as paramagnetic relaxation enhancement experiments by our group, which showed that the cytosolic N-terminal domain interacts directly with the lipid bilayer in liposomes (Carl Öster *et al.*, manuscript in preparation). Overall, these findings indicate that the N-terminal–deficient GlpG has a reduced ability to remodel the PC-loaded lipid bilayer, leading to a non-native reorientation and modified substrate interaction than the wildtype GlpG.

Separate work has revealed lipid dependence for native-like activity in other rhomboid members. It has been shown that PARL cleavage is faster in the presence of cardiolipin (114), and human protease RHBDL4 activity is regulated by cholesterol levels *via* direct and specific protein–lipid interaction through the conserved GxxxG motif (115). A recent article has also shown that RHBDL4 activity is regulated by the degree of membrane saturation at the ER, with differential cleavage positions for both amyloid precursor protein (115) and SREBP-1c (100). Taken together with the work on membrane thinning requiring interaction with PE or PG lipids, this points to a fundamental role in membrane sensing for rhomboid protease function and disease states, as well as a wider dependence of the lipid environment for regulation of rhomboid members that requires further work.

The recent experimental work on membrane thinning specifically has partially answered a long-standing question: Why are inactive rhomboids so prevalent, necessary, and conserved? We now know that conserved rhomboid features generate local membrane thinning, and in relation to ERAD, the thinning provided by Derlins allows for a reduced energy barrier to poor-quality membrane protein extraction from the ER for degradation (61, 62, 116, 117). However, this is not the complete picture of the function of pseudoproteases: It has also been shown that Derlins are responsible for initial recognition of ERAD clients (6), can modulate ERAD machinery composition in response to ER stress (19), and can even perform chaperone functions for misfolded membrane proteins using this lipid thinning independent of ERAD (20), indicating that ERAD-related pseudoprotease function is but one aspect of their functionality in evolution.

## Challenges and outlook

The level of knowledge gained about the structure and function of rhomboids since their initial discovery nearly a quarter of a century ago is remarkable. Initial publications examining protease structure and function *in vitro* have laid the initial groundwork for examination of physiological function, substrate recognition, and cleavage mechanisms of proteolytically active rhomboid members. This is now increasingly being combined with studies of rhomboid proteins *in vivo*, and the crosstalk between researchers of both active and inactive proteases has accelerated shared understanding of this family in biological context—in particular relating to the rhomboid fold’s interaction with the membrane.

However, more work is always required. Biochemically, progress has been made to develop inhibitors of active protease members, but specificity within such a common fold proves challenging and a matter of ongoing research (*e.g.*, see our proof-of-principle study of inhibitor binding to GlpG by ssNMR (118)). Notably, there is a distinct lack of high-resolution structures of various rhomboid members (currently five of the hundreds of thousands of identified members), which is needed not only for inhibitor design but also to better inform how such diverse roles are mediated between similarly structured proteins, often within the same organism and even membrane within an organism. The use of predictive structural algorithms such as AlphaFold (119) to produce some of these structures is tempting, especially for such a conserved structural fold; however, caution must be taken to not overgeneralize members between one another. In addition, whilst AlphaFold and other predictive structural algorithms are a powerful tool, the reliance on structures from the Protein Data Bank, which (so far) has a majority of soluble proteins, leads to difficulties with integral membrane protein structure prediction, the specific dynamics membrane proteins are accustomed to, and in general do not account for other vital lipid–protein interactions that are necessary for a full understanding of rhomboid protein’s structure and dynamics.

There are several questions remaining of how rhomboids distinguish substrates from nonsubstrates, and structural and dynamic information of this interrogation in the native membrane environment is missing. This is likely to be studied in more detail in the near future, since the importance of the lipid environment on the rhomboid family has been highlighted in recent publications, and techniques have become available to examine protein structure and function within these native environments, such as ssNMR and cryo-EM. These will likely prove very fruitful to our holistic understanding of the rhomboid family as well as specific rhomboid members in the future.

## Data availability

The data used for this review (*i.e*., protein sequences and their subsequent alignment) can be requested through sawczyc@fmp-berlin.de. UniProt accession codes used to generate Figure 1 are provided in Table 1.

## Conflict of interest

The authors declare that they have no conflicts of interest with the contents of this article.
